# Thermoregulatory roles of preoptic area histamine H _1_ receptor-expressing neurons

**DOI:** 10.64898/2026.09.22.753241

**Published:** 2026-09-28

**Authors:** Ramón A. Piñol, Arianna Valeri, Cuiying Xiao, Colleen K. Hadley, Abigail I. Goldschmidt, Damien Kerspern, Soumya Kulkarni, Oksana Gavrilova, Andrew Lutas, Michael J. Krashes, Marc L. Reitman

## Abstract

The preoptic area (POA) is a key regulator of body temperature, orchestrating behavioral and physiological thermoregulatory functions, including fever, torpor, and homeostatic responses to ambient temperature. Histamine regulates body temperature across animal taxa, including mammals, lower vertebrates, and invertebrates. *Hrh1* , which encodes the histamine H _1_ receptor, is expressed in several neuronal populations in the POA with limited overlap with other POA thermoregulatory neurons. Acute activation of POA ^Hrh1^ neurons increased body temperature and physical activity. Acute inhibition attenuated the dark phase increase in body temperature without affecting physical activity. There was no effect of inhibition on body temperature during the light phase, fever, cold exposure, or warm exposure. Assessing behavioral thermoregulation, we found that POA ^Hrh1^ neurons stimulated nest building in both sexes, possibly contributing to cold-induced nesting behavior. Through projections to the arcuate, dorsomedial hypothalamic and tuberomammillary nuclei, POA ^Hrh1^ neurons can increase body temperature and physical activity. Overall, POA ^Hrh1^ neurons participate in several thermoregulatory functions, including daily body temperature rhythm regulation, physical activity, and nest building behavior.

## Full Text Availability

The license terms selected by the author(s) for this preprint version do not permit archiving in PMC. The full text is available from the preprint server.

